# Effects of a Diet Containing Sources of Prebiotics and Probiotics and Modification of the Gut Microbiota on the Reduction of Body Fat

**DOI:** 10.3390/ijerph20021348

**Published:** 2023-01-11

**Authors:** Paweł Jagielski, Izabela Bolesławska, Iwona Wybrańska, Juliusz Przysławski, Edyta Łuszczki

**Affiliations:** 1 Department of Nutrition and Drug Research, Institute of Public Health, Faculty of Health Sciences, Jagiellonian University Medical College, 31-066 Kraków, Poland; 2 Department of Bromatology, Poznan University of Medical Sciences, 60-806 Poznań, Poland; 3 Clinical Biochemistry, Department of Genetics and Nutrigenomics, Faculty of Medicine, Jagiellonian University Medical College, 31-501 Kraków, Poland; 4 Institute of Health Sciences, Medical College of Rzeszów University, 35-310 Rzeszów, Poland

**Keywords:** obesity, eating behaviors, prebiotics, probiotics, gut microbiota

## Abstract

In 2022, according to the World Health Organization (WHO) report, overweight and obesity have reached epidemic proportions in the WHO European Region, affecting almost 60% of adults. Based on the assessment of BMI (Body Mass Index), a group of 56 women aged 25–45 years (31 women group A average BMI 34.9 ± 4.86 kg/m^2^ and 25 women group B average BMI 33.4 ± 4.02 kg/m^2^) were qualified for the study. In a multi-center, two-arm, parallel, non-randomized study, two types of weight-reduction diets (A and B) were used over a 3-month period. In group A, a standard low-energy diet was used with individually adjusted caloric intake of 1100–1300 kcal, with an increase in the amount and frequency of consumption of sauerkraut and groats and a daily intake of fermented milk drinks (300–400 g), fermented cucumbers (100 g), mineral water (1 L) and cod liver oil (5 mL). In group B, a standard low-energy diet with individually adjusted caloric intake of 1100–1300 kcal with daily intake of fermented milk products (150 g), highly mineralized water (0.5 L), once a week fermented cucumbers, and once a week buckwheat groats was used. The following measurements were taken: body weight, body fat mass, water content, body height, waist circumference, and hip circumference. Body weight and body composition were measured using the Tanita MC-780 MA and TANITA BC-601 analyzer using the bioelectric bioimpedance method. The stool samples were analyzed in the microbiology laboratory where quantification of *Bifidobcaterium* spp., *Bacteroides* spp., *Faecalibacterium prausnitzii* species, *Akkermansia muciniphila* and total bacterial count (TBC) was performed. Under the influence of the introduced nutritional intervention, a statistically significant reduction in body weight, body fat, waist circumference, and hip circumference was demonstrated after 3 months. Under the influence of weight reduction, as well as dietary changes, there was an increase in the number of *Akkermansia muciniphila* bacteria in the women studied. The low-energy diet containing sources of natural prebiotics and probiotics had a more favorable effect on the number of *Faecalibacterium prausnitzii* bacteria compared to the standard diet.

## 1. Introduction

Obesity is a chronic disease characterized [1] by abnormal or excessive fat accumulation that can impair health. It is diagnosed when the BMI (Body Mass Index) exceeds 30 kg/m^2^ [2].

In 2022, according to the World Health Organization (WHO) report, overweight and obesity have reached epidemic proportions in the WHO European Region, affecting almost 60% of adults [3]. Children are also affected, with 7.9% of children under 5 years of age and one in three school age children living with obesity or being overweight [4]. In Poland, according to WHO data, in 2016, 65.6% of men and 51.1% of women were overweight, of which obesity was present in 22.2% of women and 22.7% of men [2].

Obesity is a cause of numerous diseases, including type 2 diabetes, cardiovascular disease, malignant tumors, gallstones, nonalcoholic steatohepatitis, endocrine disorders, or osteoarthritic changes [5]. It is also the leading risk factor for disability, causing 7% of all years of life with disability, and is associated with increased morbidity and mortality from COVID-19 [4].

It is estimated that the fight against obesity and the health problems it causes in 2015 consumed around 14 billion PLN from the Polish health budget, which represented 20% of its total [6]. According to a 2015 report by the Mc Kinsey Institute, worldwide obesity generates a total cost of up to $2 trillion per year [7]. Addressing obesity is critical to achieving the Sustainable Development Goals and is a priority in the European Programme of Work 2020–2025. United Action for Better Health [4].

Obesity develops throughout life through two mechanisms: developmental programming based on preconception and gestational exposure to obesity, unhealthy diet, and physical inactivity [8,9,10]

Obesity can also be influenced by the gut microbiota—the effects of gut microbes on the production of short-chain fatty acids, which in turn affect the homeostasis of the body’s energy are being studied [7,11].

The gut microbiota of adult individuals consists mainly of bacteria belonging to two types: *Bacteroidetes* and *Firmicutes* [12]. Numerous studies show that the ratio of these bacteria is different in obese compared to normal-weight individuals [13]; however, there are also studies that do not confirm the relationship between the *Bacteroidetes/Firmicutes* ratio, diet, and body mass index (BMI) [7].

Fermented dairy products, sauerkraut, and fermented cucumbers are potential sources of probiotic microorganisms that can have a beneficial effect on the gut microbiota [14]. However, published results in this area are inconclusive and require further research.

Buckwheat groats, lentils, and millet groats are sources of potential prebiotics, that is, non-digested food components with beneficial effects on host health due to modification of the intestinal microbial complex [15,16,17]. Studies show that the fiber fractions contained in buckwheat, millet and lentils have a beneficial effect on the composition of the intestinal microbiota, as well as the bioactive components produced by bacteria [18,19].

Fermentation is a traditional and popular method of food preservation in Poland [20]. Fermented cucumbers and cabbage are most commonly consumed in this form [21]. Fermented dairy products are also common in the diet of Poles. In the past, they were mainly consumed in the form of soured milk [22] and kefir [23] and now yoghurts are increasing in popularity [24]. Poland is also the world’s leading producer of buckwheat [25,26], consumed mainly in the form of groats [27].

Given that the fight against obesity is still not producing the expected results, more research needs to be done that takes into account a multifactorial approach to such a complex problem as obesity.

The purpose of this study was to investigate the effect of diet containing sources of prebiotics and probiotics on the modification of the gut microbiota and on the reduction of body fat in a group of obese adults.

## 2. Materials and Methods

A multicenter, 3-month, two-arm, parallel, nonrandomized study on the evaluation of the effectiveness of a low-calorie diet enriched with potential sources of prebiotics and probiotics in reducing body fat compared to a standard reduction diet was conducted according to the guidelines of the Declaration of Helsinki. The study received positive approval from the Bioethics Committee of the Jagiellonian University (KBET/96/B/2014 26 June 2014).

The study participants were introduced to the purpose of the study. They were also informed how the study would be conducted. Consent was obtained from all participants to participate in the study.

### 2.1. Patients

The study was carried out simultaneously in two centers at the Institute of Public Health of the Jagiellonian University Medical College in Krakow (group A) and the Karol Marcinkowski Medical University in Poznan (group B) according to identical protocols. Potential participants in the study were recruited through advertisements on social networks and by the snowball method. Based on the assessment of BMI, a group of 56 women aged 25–45 years (31 women group A and 25 women group B) were qualified for the study. There was no significant age difference between the groups. All women who agreed to participate in the study were examined by a qualified dietitian during the inclusion visit to meet the protocol requirements.

Inclusion criteria included female sex, absence of comorbidities, presence of simple obesity assessed by BMI > 30 kg/m^2^ or BMI > 29.0 kg/m^2^ and body fat score (BF%) that indicates obesity (>30%). A condition for inclusion in the study was also the non-use of antibiotics and probiotic/prebiotic supplements 3 months prior to participation in the study. Inclusion of these during the study was also associated with exclusion from the study.

Pregnant or breastfeeding women, those with metabolic diseases, those on another reducing or therapeutic diets, those with allergies/hypersensitivity to the products used in the diet were excluded.

Taking into account the individual variability of the implementation of dietary recommendations versus the dietary advice given to study participants, the classification of women into diet groups A and B was verified using two-stage cluster analysis. The variables of intake after 3 months were entered into the model: groats, sauerkraut, highly mineralized waters and fermented dairy products variables that were assumed to differentiate the two groups. A person on diet B was classified as meeting the assumptions of diet A, while three people on diet A were classified as meeting the assumptions of diet B. By using cluster analysis and correction, each of the resulting groups became more homogeneous, while at the same time, the variation between groups increased. In the end, the size of the group following diet A was 19 people, while diet B was 23 people. The aforementioned classification adjustment did not affect the changes in anthropometric parameters compared to the initial groups of 21 subjects each. Of the 56 people included in the intervention, 10 women dropped out of group A and 4 women in group B, 21 women in group A and 21 women in group B completed the reduction diet. No side effects or adverse events were reported.

### 2.2. Study Design

In both study groups, after a medical evaluation of body weight and health risks of introducing dietary intervention, a qualified dietitian established a weight reduction diet with a fixed caloric intake of 1100–1300 kcal.

In both groups, a standard low-energy diet was used according to the key guidelines for the dietary treatment of obesity [28] and the 2015 Polish Dietetic Association “Standards for the dietary treatment of simple obesity in adults—PTD Position Statement 2015” [29] and the current dietary recommendations of the National Center for Nutrition Education as presented in the Pyramid of Healthy Eating and Physical Activity for Adults and the Principles of Healthy Eating [30]. In addition, a higher frequency and amount of consumption of potential prebiotic and probiotics was introduced in Group A.

The duration of the intervention was 3 months and occurred simultaneously in both study groups. All measurements and their analysis were carried out at the University of Medicine in Poznan and the Institute of Public Health of the Jagiellonian University Medical College in Krakow before and after each intervention period. All outcomes were evaluated using identical methods in both study groups.

Follow-up meetings with the dietitian were held twice a month to verify compliance with the study protocol.

This study presents the results of endpoints such as analysis of the gut microbiota, anthropometric measurements, analysis of body composition and analysis of food intake before and after the intervention.

### 2.3. Dietary Intervention

The diet intervention was determined for each patient individually by a certified dietitian according to current guidelines for the dietary treatment of obesity [28,29].

An individual assessment of the calorie content of the diet prior to the start of the study was made based on the results obtained from the current recording of whole food rations carried out over 2 weekdays and 1 weekend day (3 days). These data were entered into the Diet 5.0 program, which enabled the calculation of the current calorie content of the diets of the study participants.

In accordance with the current ESPEN recommendations [31] for the management of weight loss in female participants, a reduction in the calorific value of the diet of 700 kcal was introduced, resulting in diets with an average calorific value of 1100–1300 kcal.

In group A, a standard low-energy diet with an increase in the amount and frequency of consumption of sauerkraut and groats and a daily intake of fermented milk drinks (300–400 g), fermented cucumbers (100 g), mineral water (1 L) and cod liver oil (5 mL) was used.

In group B, a standard low-energy diet with daily intake of fermented milk products (150 g), highly mineralized water (0.5 L), once a week fermented cucumbers, and once a week buckwheat groats was used. Detailed characteristics of diets A and B are shown in Table 1.

### 2.4. Experimental Procedure

#### 2.4.1. Dietary Assessment

Dietary assessment before and after the intervention was based on the analysis of food diaries obtained by the method of current recording of product intake, carried out in accordance with the guidelines of the Committee on Human Nutrition of the Polish Academy of Sciences in Warsaw [32] and the Best Practice Guidelines (BPGs) [33]. The study was carried out systemically, that is, the product and food intake diary obtained from each participant included 3 days (2 working days and 1 weekend day) before starting the diet and 3 days (2 working days and 1 weekend day) after 3 months on the diet. To determine the amount of food and dishes consumed, study women were asked to record the weight of the products using kitchen scales or by using the website http://www.ilewazy.pl [34], which contains a database of photographed and weighed products and dishes. Alternatively, the women surveyed used household measures (plate, glass, spoon, slice). The diet interviews obtained were verified by a certified dietitian, and the weights of the products and dishes consumed were clarified using the photo database from the “Photo album and products and dishes” [35].

The analysis of the results of the questionnaires on the qualitative and quantitative composition of the whole day rations was carried out using computer databases based on the “Tables of composition and nutritional value of food” [36]. Assessment of the level of intake of individual nutrients was carried out in an application in Diet 5.0 software [37].

#### 2.4.2. Anthropometric Measurements

Each participant in the study had body composition analyzed twice—before the intervention and after 3 months of intervention. The following measurements were taken: body weight, body fat mass, water content, body height, waist circumference, and hip circumference. Body weight and body composition were measured using the Tanita MC-780 MA and TANITA BC-601 analyzer using the bioelectric bioimpedance method. This method involves measuring the resistance represented by individual tissues to an electrical impulse that affects them. Adipose tissue shows greater resistance to the impulse, while water conducts electrical impulses well. A centimeter tape graduated to 1 mm was used to assess waist and hip circumference. Participants’ body height was measured using the legalized telescopic height gauge SECA 799.

The study was carried out according to the current methodology at the same time of day, with a minimum interval of 3 h after the last meal and exercise. Anthropometric evaluation was performed identically in both study groups. Measurements were taken in a standing position, barefoot, with the legs slightly apart and the arms spread [38].

#### 2.4.3. Analysis of the Intestinal Microbiota

The analysis of the intestinal microbiota was carried out in stool samples, which were provided by the study participants in special sterile containers. Study participants were informed in advance of the stool collection method for the study. The stool samples were collected from the study participants on the same day and frozen at −20 °C. Then, while maintaining the same temperature, the stool samples were transferred to the Institute of Microecology in Poznań where quantification of *Bifidobcaterium* spp., *Bacteroides* spp., *Faecalibacterium prausnitzii* species, *Akkermansia muciniphila* and total bacterial count (TBC) was performed.

The tests were carried out using the 7300 Real-Time PCR System, from Applied Biosystem (Thermo Fisher Scientific, Waltham, MA, United States of America) by PCR. DNA for the assay was isolated from 180–220 mg of feces. The QIAamp Fast Stool Mini Kit DNA (Qiagen) was used for DNA isolation.

#### 2.4.4. Statistical Analysis of the Data

The results obtained were entered into a database in a Microsoft Excel spreadsheet and analyzed using IBM SPSS Statistics 24 and STATISTICA 12.0 PL software (UJ licenses). The normality of the distribution of the quantitative variables analyzed was checked using the W Shapiro–Wilk test. Depending on the result of the normality of distribution analysis, parametric or nonparametric statistical methods were applied. The results for quantitative variables with a normal distribution were presented as the arithmetic mean (X) ± standard deviation (SD), while for variables with a non-normal distribution or for ordinal variables, the results were presented as the median (Me) ± quartile deviation (Q). Homogeneity of the study groups was checked using the Student’s *t* test or its nonparametric equivalent, the Mann–Whitney U test, prior to the study. The occurrence of an interaction between diet type and duration of dietary intake was tested using ANOVA analysis for repeated measures with interaction. If there was no interaction between the type of diet and the time, the *p*-value for the interaction and the change over time was presented for the total subjects. If there was an interaction, two p-values calculated for both diets were presented based on an ANOVA model with repeated measures for each diet separately. When the assumptions of using ANOVA analysis for repeated measures were not met, it was first checked with the Mann–Whitney U test to see if there were differences between changes over time by diet type and then with the Wilcoxon test to see if changes were statistically significantly different over time. To more accurately classify the study women into diet groups A or B, a two-stage cluster analysis was performed. The level of statistical significance was taken as α = 0.05 [39].

## 3. Results

### 3.1. Characteristics of the Study Group

Before the intervention, the study groups of women (A and B) did not differ statistically significantly in terms of age (the mean age of the study women was 37.26 ± 8.46 years), educational level (half of the study women had a university degree), occupational status (21 of the study women were white-collar workers), marital status (24 of the study women were married) and physical activity (24 of the study women declared that they did not exercise) (*p* > 0.05). Detailed data are presented in Table S1, Supplementary Materials.

### 3.2. Analysis of Food Product Intake before and after the Intervention

Before the study, the median contents of the food products analyzed in the diets of women in groups A and B were not significantly different (*p* > 0.05) (Table S2, Supplementary Materials).

After 3 months of following the diet in both groups (with no statistically significant differences between diets A and B), there was a significant increase in the intake of fermented cucumbers (*p* < 0.0001), vegetables (*p* < 0.0001), total vegetables and fruit (*p* < 0.0001) and fish (*p* = 0.0111).

Adherence to the diet also resulted in a significant increase in cereal intake, all fermented milk drinks, kefir and highly mineralized water in group A and an increase in fruit intake, fermented milk drinks, high mineralized water and a decrease in coffee intake in group B (*p* < 0.05).

After 3 months of dietary intake, the rations of the women of group A showed a statistically significantly higher intake of highly mineralized water than those of the women of group B (*p* < 0.0001) as well as fermented milk products (*p* = 0.0039), cereals (*p* < 0.0001) and kefir (*p* = 0.0006). On the contrary, there were significantly more fruits (*p* = 0.0013) and less coffee (*p* = 0.0173) in the rations of the women of group B. The results are shown in Table 2.

### 3.3. Assessment of Changes in Anthropometric Parameters

Before the intervention, the study groups of women (A and B) did not differ statistically significantly in height and weight, BMI, Waist to Hip Ratio (WHR), body fat (BF), total body water (TBW) and waist and hip circumference (*p* > 0.05).

After 3 months of diet, there were favorable changes in body composition parameters in the women studied. The type of diet used did not affect the changes. The only difference was observed in the WHR index, with only diet A causing a significant decrease (Table 3).

After 3 months of following a diet, regardless of the type of diet, there was a reduction in body weight (*p* < 0.0001), fat mass (*p* < 0.0001), waist circumference (*p* < 0.0001), and BMI (*p* < 0.0001) in both study groups.

The mean percentage reduction in body weight was 7.37 ± 4.49%, while the mean percentage reduction in body fat was 15.62 ± 9.04%. The relative reduction in body weight in almost 65% of the women studied was above 5%, while the relative reduction in body fat in more than 65% of the women was above 10%.

**Figure 1:**
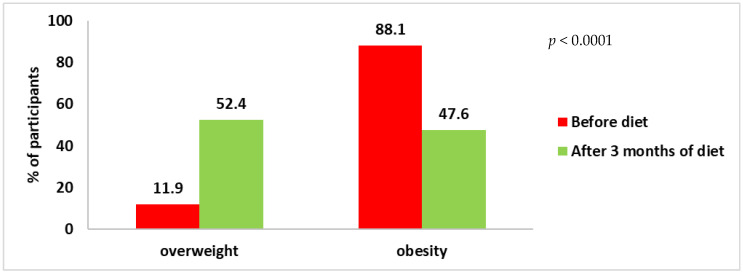
Classification of total female subjects according to BMI.

**Figure 2:**
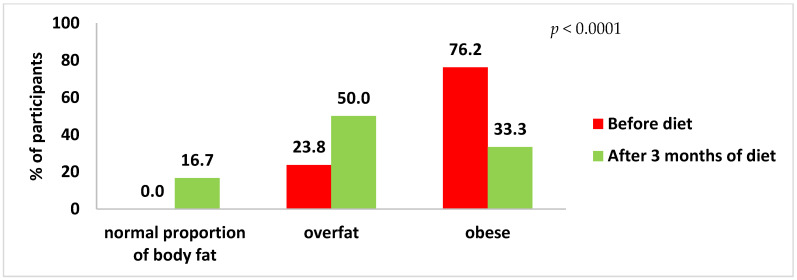
Classification of total female subjects according to body fat content.

Hip circumference decreased more in women on diet B compared to women on diet A (8.17 ± 4.86 cm vs. 5.37 ± 3.74 cm) and in both cases these changes were statistically significant (*p* < 0.0001). Before the intervention, 88.1% of the women were obese, while only 47.6% were obese after three months on the diet (*p* < 0.0001) (Figure 1). For the classification based on body fat measurement, the percentage of obese women decreased from 76.2 to 33.3% after 3 months (*p* < 0.0001) (Figure 2).

### 3.4. Assessment of Changes in the Intestinal Microbiota

Before the study, the number of *Bifidobacterium* spp., *Bacteroides* spp., *F. prausnitzii* species, *A. muciniphila* species, and the total number of bacteria in 1 g of feces did not differ between groups A and B of women.

**Figure 3:**
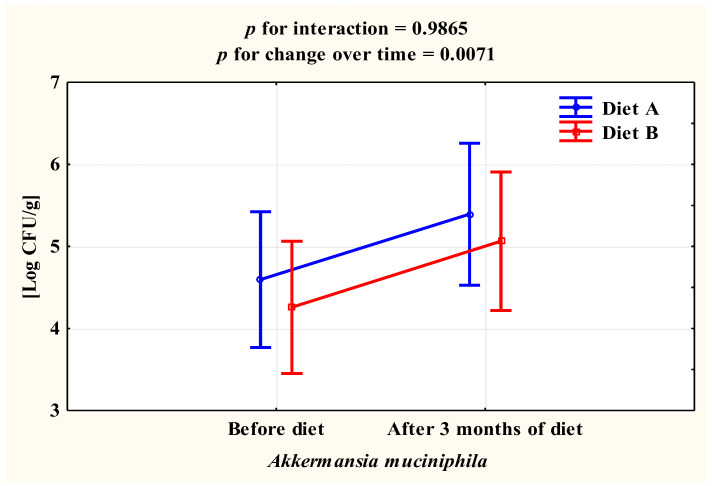
Change in the number of *A. muciniphila* in the stool samples of the women studied.

After 3 months on the diet, the number of *Bifidobacterium spp.* did not change. There were also no statistically significant differences according to the type of diet used. In the case of *Bacteroides spp.* bacteria, there was an increase in their number in women following diet A by 0.25 ± 0.43 log CFU/g (*p* = 0.0200), while no change was found in women following diet B (*p* > 0.05). For the total bacterial count, women on diet B showed a statistically significant reduction of 0.28 ± 0.46 log CFU/g (*p* = 0.0126), while no change was found in women on diet A (*p* > 0.05). In the case of the bacteria *A. muciniphila,* there was a statistically significant increase in both study groups of 0.80 ± 1.73 log CFU/g (*p* = 0.0071) (Figure 3). In the case of bacteria of *F. prausnitzii,* there was a statistically significant difference between the type of diet used. Among women on diet A, the number of these bacteria did not change after 3 months (*p* > 0.05), whereas there was a statistically significant reduction of 0.41 ± 0.65 log CFU/g (*p* = 0.0102) in women on diet B (Table 4).

## 4. Discussion

The study groups of women (A and B) had similar age, education level, occupational status, marital status, and physical activity before the intervention. Both study groups of women also had similar mean values of anthropometric parameters, BMI was indicative of first-degree obesity (30–34.9 kg/m^2^) [40], the % fat content was approximately 10% above the threshold value for the diagnosis of obesity of 30% and the waist circumference indicated central fat accumulation associated with a high risk of comorbidities [41].

One reason for the prevalence of obesity was low physical activity [42], as many as 53% of women in group A and 65% of women in group B declared complete lack of physical activity. In the diets of both studied groups of women, a high frequency of consumption of white bread, meat and processed foods, sugar and sweets was observed, and a very low content of whole-meal bread, groats, fish, dry pulses, and highly mineralized water, with low vegetables and fruits (an average of 200 g of vegetables and 100 g of fruits). Adherence to this type of diet (the so-called western diet) rich in processed foods, red meat, sugar, and fat is associated with increased rates of obesity [43,44,45]. On the contrary, the use of diets high in fruits, vegetables, unprocessed cereals, legumes, nuts, and seeds is inversely associated with the risk of developing obesity [46,47,48,49].

Dietary interventions that induce a negative energy balance are the cornerstone of overweight and obesity treatment and are part of the standard recommendation [41]. To achieve weight reduction, we used a standard dietary approach based on a low-energy diet (diet B) and a low-energy diet supplemented with fermented dairy drinks, fermented vegetables (sauerkraut and fermented cucumbers), buckwheat groats, millet groats or lentils, cod liver oil and highly mineralized water (group A). The selection of products in group A was mainly due to their beneficial role in the prevention and treatment of obesity, but also due to their ease of introduction into a weight loss diet due to the fact that they are a common component of traditional meals.

Numerous animal studies indicate the potential of omega-3 polyunsaturated fatty acids (PUFAs) against obesity by reducing body weight, fat deposit mass, and adipocyte size, as well as reducing food intake and influencing the microbiome [50,51,52,53]. In addition, the n-3 PUFAs contained in trans can be considered prebiotics due to their ability to increase the production of anti-inflammatory short-chain fatty acids [54].

Consumption of buckwheat (*Fagopyrum esculentum*) reduces BMI and body fat mass [55] changes in postprandial gastrointestinal satiety levels [56] ability to shorten intestinal transit time [57] and has influences on the composition of the microbiome [58]. As a source of prebiotic carbohydrates, including resistant starch and other types of indigestible starch [17], lentils are also a potential dietary component to reduce obesity. Numerous studies indicate the beneficial effects of highly mineralized water in the prevention and treatment of obesity by inhibiting lipid accumulation, reducing fat mass and adipocyte size, and regulating lipid metabolism [59,60,61].

Several studies confirm beneficial effects of fermented milk drinks on fat reduction and on appetite regulation [62,63]. Although for sauerkraut and cucumbers there is no direct evidence of beneficial effects in the prevention or treatment of obesity, they exert a beneficial effect on the microbiota [64]. They are a potential source of Lactobacillus strains with putative probiotic potential [65,66].

A dietary intervention based on a standard reducing diet (group B) and a diet enriched with sources of prebiotics and probiotics (group A) resulted, after 3 months, in both groups of obese women in a favorable increase in the dietary intake of fermented cucumbers, vegetables, fermented beverages and highly mineralized water, vegetables and fruit and fish. At the same time, after the introduction of the dietary intervention, a higher intake of cereals and kefirs was found in group A and fruit in group B. The women in group A also had a higher intake of highly mineralized water and fermented dairy products than the women’s rations in group B. Therefore, it seems that the consumption pattern in group A was more conducive to weight reduction than in group B, mainly due to a higher dietary supply of fermented dairy products and highly mineralized water.

The main goal of a weight loss diet is to reduce body fat. The introduction of both diets resulted, similar to other authors [67,68,69,70,71], in a reduction of more than 5% in body weight in approximately 63.3% of women in both groups, body fat mass (by about 16 per cent) and waist circumference and BMI. In our study, hip circumference decreased in both study groups, but, interestingly, more in women following diet B than diet A (8.17 ± 4.86 cm vs. 5.37 ± 3.74 cm).

After 3 months of dietary intervention, the percentage of women with first-degree obesity (assessed by BMI) decreased from 52.4% to 23.8%.

Interestingly, after 3 months of intervention, both diets resulted in a similar effect, and we found no significant differences in body weight, body fat, or waist circumference reduction between women following diet A and those following diet B. This shows that it was not so much the increased intake of fermented milk drinks, fermented vegetables, cereals, lentils, cod liver oil, and highly mineralized water (group A) that had a positive effect on weight reduction and improvement in anthropometric parameters, but rather the reduction in the energy value of the diet, the introduction of regularity of meal intake, more whole grain products and vegetables and fruit (approximately 600 g/day).

No impact of pre- and probiotic food sources (yoghurt, oligofructose) on weight loss has also been shown in studies by other authors [62,72]. A study by Sanchez et al. [73] observed no statistically significantly difference between the groups of obese women taking the probiotic containing *Lactobacillus rhamnosus* after 12 weeks and only after 24 weeks. Therefore, it is possible that the duration of the intervention was too short to conclude that there was a significant difference in the effect of a diet enriched in pre- and probiotic sources (diet A) compared to the typical reduction diet (diet B). It is also possible that the amounts of prebiotics and probiotics consumed in diet A was too small to produce a significant difference with diet B, which also contained synbiotic components in whole grain cereals, dark rice, bran, flaxseed, chia seeds, fish and vegetables, fruit and, although in much smaller amounts, in lactofermented foods and fermented vegetables.

Numerous lines of evidence support the involvement of the gut microbiota in the initiation and development of obesity and related diseases [74,75,76,77]. The composition of the gut microbiota differs in obese and lean individuals [78], suggesting that the dysbiosis of the microbiota may contribute to weight changes [76]. Administration of prebiotics, probiotics, and postbiotics reverses intestinal dysbiosis [79] affecting body weight and body composition.

In our study, before the introduction of the diet intervention, the number of *Bifidobacterium* spp., *Bacteroides* spp., *Faecalibacterium prausnitzii* species (*F. prausnitzii*), *Akkermansia muciniphila* (*A. muciniphila*) species and the total number of bacteria in 1 g of feces were similar in women on diets A and B.

After 3 months of intervention, there was no effect of diet type on the number of *Bifidobacterium* spp. However, the number of *Bacteroides* spp. increased in the group of women on diet A, which is favorable given that a negative correlation was found between the number of *Bacteroides* and body weight, BMI and body fat [75,80,81]. An increase in *Bacteroides* counts was observed under the influence of whole grain cereals [82] and following a low-calorie diet [83]. However, the use of a low-calorie diet for 8 weeks in obese men did not change the number of *Bacteroides* [84] as in our study in group B (diet with less pre- and probiotics).

At the same time, the total number of bacteria decreased after the B diet. In a study by Palmas et al. [81], lower microbial richness and diversity were associated with obesity, so the reduction in body weight observed in our study should instead result in increased microbial richness.

The total number of *F. prausnitzii*, which is one of the main producers of butyrate in the intestine and plays a key role in intestinal physiology and host well-being through its anti-inflammatory effects, also decreased in the B-diet group of women after 3 months [85,86]. However, the relationship between obesity and the abundance of *F. prausnitzii* in the human colon is currently unclear [87] and decreases after a low-calorie diet [84]. A low mean number of *F. prausnitzii* was also observed in patients with Crohn’s disease [88], with infectious colitis [89] and in diabetic patients [90]. However, given that resistant starch [91] and PUFA omega-3 [92] increase the abundance of bacteria closely related to *F. prausnitzii* among dietary factors, the low supply of starch in the diet of women in group B may have been responsible for the decrease in *F. prausnitzii* abundance. On the other hand, fish oil as a source of PUFA omega-3 may have protected the abundance of *F. prausnitzii* in group A.

Therefore, it is possible that the high amounts of prebiotics and probiotics in the diet of group A protected the abundance of *F. prausnitzii*, while diet B with fewer of these products resulted in a decrease in the abundance of *F. prausnitzii*. These results would need to be confirmed by performing an additional analysis of butyrate concentrations in fecal samples.

The changes observed in intestinal microflora after omega-3 PUFA supplementation in other studies were related to a decrease in *Faecalibacterium* [93,94], which was not observed in group A using 5 mL of trans/day.

In our study, there was an increase in *Akkermansia muciniphila* in both groups A and B after 3 months of diet and weight reduction. A similar increase in *A. muciniphila* and a decrease in body weight was observed in obese subjects with type 2 diabetes after 4 months of diet [95], while *A. muciniphila* was significantly lower in obese or overweight children [96]. Yassour et al. [97] in monozygotic twins from South Korea found that the number of *A. muciniphila* was negatively correlated with BMI.

To our knowledge, this is the first study to compare the effect of a low-calorie diet versus a low-calorie diet enriched with pre- and probiotics on weight loss and improvement in anthropometric parameters and gut microbiota composition. A limitation of the study may have been the use of the bioelectrical impedance method to assess body fat content, rather than the currently recognized ‘gold standard’ of dual energy X-ray absorptiometry (DXA). Additionally, currently available technology, smartphones, and smartphone applications were not used to monitor daily intake. Furthermore, the women in the diet A group did not increase their intake of sauerkraut and lentils as intended, which may have affected the final results. The fact that kefir, yoghurt, curdled milk, sauerkraut, and fermented cucumbers were sourced from different manufacturers may also have been a limitation, which could have been a source of additional variability. We based our assessment of the women’s health status on their verbal declaration. We did not analyze short chain fatty acids (SCFA), especially butyrate concentrations in fecal samples, which could further clarify the relationship between the intestinal microflora and the results of the diet. Another limitation of the study was the lack of randomization due to the assumption that each group would be conducted at a different center and that the study was only conducted on women, so the results cannot be extrapolated to the whole population.

## 5. Conclusions

In conclusion, our results suggest that following a low-calorie diet enriched with pre- and probiotic sources has a similar effect on reducing weight, BMI, body fat, waist circumference and hip circumference as a typical low-calorie diet.

Both dietary interventions A and B, as well as overall moderate weight loss induced by calorie restriction (in whatever form) were associated with changes in the gut microbiome of adult women with obesity. Under the influence of weight reduction, as well as dietary changes, there was an increase in the number of *Akkermansia muciniphila* bacteria in the women studied. With very favorable changes in the microbiome observed after diet A (increase in *Bacteroides* spp. protection of *F. prausnitzii* abundance). The diet without pre- and probiotics induced an unfavorable decrease in total bacterial counts and *F. prausnitzii*.

The promising effect of pre- and probiotics in a low-calorie diet and the lack of side effects encourage future research in this area. Long-term prospective studies with participants of both sexes could confirm the beneficial interaction between a low-calorie diet enriched with pre- and probiotics and the gut microbiota.

Evaluation of butyrate, the main fermentation product of *F. prausnitzii*, would determine whether a diet containing pre-, and probiotics has an anti-inflammatory effect during weight reduction. A better understanding of the relationship between the human gut microbiota and obesity and the impact of a weight loss diet on the composition of the microbiota may lead to new possibilities for the prevention and more effective treatment of obesity.

## Figures and Tables

**Table 1:** Characteristics of the intervention diets.

Intervention	DIET A	DIET B
Basic assumptions	A low-energy diet (rich in fat and simple sugars), determined individually, usually about 1100–1200 kcal. Based mainly on a high supply of complex carbohydrates (source of dietary fiber) in the form of dark bread, brown rice, groats, with the maximum elimination of simple sugars and fats, especially of animal origin, while providing protein in the amount of 1–1.5 g per kg of body weight due, in the form of lean meat, poultry, fish, and dairy products. Rich in fresh and cooked vegetables.	
Fermented dairy products (kefir, yogurt, curdled milk)	Every day 300–400 g	Every day 150 g
Fermented cucumbers	Every day 100 g	Once a week
Highly mineralized water	Every day 1000–1500 mL	Every day 500 mL (the remaining amount of fluids, i.e., 500–1000 mL from other sources)
A meal containing buckwheat groats, millet groats, or lentils	4–5 times a week 80–100 g each	Once a week 80 g
Tranium	Daily—1 teaspoon (5 mL)	-
Sauerkraut	2–3 times a week, 100 g each.	1× per week, 100 g each.

**Table 2:** Intake of selected food items by female study participants before and after 3 months of dietary change.

Products and Product Groups Analyzed	Total Respondents N = 42			Diet A N = 19			Diet B N = 23			ANOVA *p* for Interaction UMW *	ANOVA *p* for Change over TimeWilc. *
	Before	After	Change	Before	After	Change	Before	After	Change		
	X ± SD Me ± Q *	X ± SD Me ± Q *	X ± SD Me ± Q *	X ± SD Me ± Q *	X ± SD Me ± Q *	X ± SD Me ± Q *	X ± SD Me ± Q *	X ± SD Me ± Q *	X ± SD Me ± Q *		
Groats (g)	2.61 ± 7.58	24.0 ± 28.9	21.4 ± 29.2	2.00 ± 7.02	45.5 ± 28.7	43.5 ± 30.6	3.12 ± 8.13	6.29 ± 12.5	3.18 ± 7.35	**<0.0001**	**<0.0001**^A^ 0.0501 ^B^
Kefirs (g) *	0.00 ± 0.00	50.0 ± 68.8	50.0 ± 68.8	0.0 ± 0.0	210 ± 94.0	167 ± 148	0.00 ± 0.00	0.00 ± 25.0	0.00 ± 25.0	**0.0006**	**0.0010**^A^ 0.3328 ^B^
Yoghurts (g).	56.7 ± 90.5	106 ± 93.1	49.7 ± 136.4	30.8 ± 52.6	76.4 ± 108.1	45.6 ± 115	78.0 ± 109	131 ± 72.0	53.0 ± 154	0.8621	**0.0265** ^O^
Yoghurts. kefirs. soured milk (g)	71.8 ± 100	204 ± 111	132 ± 170	50.3 ± 76.5	263 ± 118	213 ± 152	89.6 ± 115	155 ± 76.8	65.5 ± 157	**0.0039**	**<0.0001** ^A^ **0.0309** ^B^
Meat. poultry. fish. hams (g)	182 ± 95.5	168 ± 53.3	−14.2 ± 109	193 ± 128	152 ± 50.0	−41.8 ± 135	173 ± 58.9	181 ± 53.2	8.50 ± 76.6	0.1373	0.3211 ^O^
Fish (g)	15.4 ± 24.4	34.9 ± 40.0	19.5 ± 45.5	25.3 ± 30.1	30.8 ± 26.3	5.50 ± 32.4	7.20 ± 14.5	38.3 ± 48.8	31.0 ± 51.9	0.0691	**0.0111** ^O^
Vegetables And Fruit (g)	351 ± 219	779 ± 301	428 ± 362	333 ± 236	654 ± 276	321 ± 368	367 ± 209	882 ± 287	515 ± 340	0.0839	**<0.0001** ^O^
Vegetables (g)	230 ± 130	587 ± 238	357 ± 281	204 ± 132	543 ± 239	339 ± 301	251 ± 127	622 ± 236	371 ± 270	0.7180	**<0.0001** ^O^
Sauerkraut (g) *	0.00 ± 1.80	0.00 ± 10.0	0.00 ± 11.7	0.00 ± 0.00	10.0 ± 15.6	26.0 ± 52.2	0.00 ± 11.1	0.00 ± 5.00	0.00 ± 16.1	0.1462	0.1586 ^O^
Fermented cucumbers (g)	12.9 ± 18.0	61.9 ± 54.7	49.0 ± 61.4	8.40 ± 11.7	68.0 ± 61.5	59.6 ± 65.5	16.7 ± 21.5	56.9 ± 49.3	40.3 ± 57.7	0.3166	**<0.0001** ^O^
Fruit (g)	122 ± 139	193 ± 146	70.8 ± 170	129 ± 143	111 ± 84.5	−17.9 ± 120	116 ± 139	260 ± 153	144 ± 173	**0.0013**	0.5246 ^A^ **0.0006** ^B^
Coffee. sugar-free infusion (mL)	324 ± 195	250 ± 173	−73.6 ± 250	250 ± 154	280 ± 147	29.4 ± 125	384 ± 207	226 ± 192	−159 ± 295	**0.0134**	0.3175 ^A^ **0.0173** ^B^
Tea (mL)	259 ± 238	263 ± 232	3.70 ± 305	208 ± 255	260 ± 253	51.2 ± 388	301 ± 219	265 ± 219	−35.5 ± 216	0.3655	0.8698 ^O^
Highly mineralized water (mL) *	0.00 ± 0.00	667 ± 458	667 ± 533	00 ± 0.00	1167 ± 355	1132 ± 610	0.00 ± 0.00	500 ± 333	217 ± 333	**<0.0001**	**0.0002** ^A^ **0.0015** ^B^

N—Number, X—Mean, SD—Standard deviation, Me—Median, Q—Quartile deviation, UMW—Mann–Whitney U test, Wilc. Wilcoxon signed-rank test, ^O^—Value of the statistic for the total subjects, ^A^—Value of the statistic for diet A, ^B^—Value of the statistic for diet B, * distribution other than normal. Bold values denote statistical significance at the *p* < 0.05 level.

**Table 3:** Anthropometric parameters of study participants before and after 3 months of diet change.

Analyzed Parameter	Total Respondents N = 42			Diet A N = 19			Diet B N = 23			*p* between the Groups before the Intervention	ANOVA *p* for Interaction	ANOVA *p* for Change over Time
	Before	After 3 Months	Change	Before	After 3 Months	Change	Before	After 3 Months	Change			
	X ± SD	X ± SD	X ± SD	X ± SD	X ± SD	X ± SD	X ± SD	X ± SD	X ± SD			
Body weight (kg)	91.8 ± 13.6	85.2 ± 14.7	−6.56 ± 4.18	93.8 ± 14.1	87.3 ± 15.1	−6.42 ± 3.51	90.2 ± 13.3	83.5 ± 14.4	−6.67 ± 4.75	0.4008 S	0.8508	**<0.0001** ^O^
BMI (kg/m^2^)	34.1 ± 4.43	31.6 ± 4.77	−2.46 ± 1.52	34.9 ± 4.86	32.5 ± 5.19	−2.42 ± 1.30	33.4 ± 4.02	31.0 ± 4.37	−2.50 ± 1.71	0.3244 U	0.8683	**<0.0001** ^O^
BF (kg)	38.5 ± 9.27	32.9 ± 10.2	−5.63 ± 3.10	40.4 ± 9.70	35.2 ± 10.4	−5.22 ± 2.76	37.0 ± 8.81	31.0 ± 9.91	−5.98 ± 3.37	0.2362 S	0.4337	**<0.0001** ^O^
BF (%)	41.5 ± 4.24	37.8 ± 5.66	−3.66 ± 2.43	42.6 ± 4.12	39.6 ± 5.02	−3.01 ± 1.91	40.6 ± 4.20	36.4 ± 5.84	−4.19 ± 2.71	0.1243 S	0.1175	**<0.0001** ^O^
TBW (%)	43.1 ± 2.80	46.0 ± 3.54	2.92 ± 1.47	43.0 ± 2.58	45.5 ± 3.31	2.52 ± 1.51	43.1 ± 3.02	46.4 ± 3.75	3.28 ± 1.38	0.9389 S	0.0957	**<0.0001** ^O^
Waist circumference (cm)	105 ± 10.9	96.4 ± 13.1	−8.15 ± 4.80	107 ± 8.87	98.1 ± 11.0	−8.53 ± 4.67	103 ± 12.2	94.9 ± 14.7	−7.85 ± 4.99	0.2556 S	0.6542	**<0.0001** ^O^
Hip circumference (cm)	119 ± 8.94	112 ± 10.2	−6.90 ± 4.56	119 ± 9.25	114 ± 10.7	−5.37 ± 3.74	119 ± 8.90	111 ± 9.83	−8.17 ± 4.86	0.9974 S	**0.0459**	**<0.0001** ^A^ **<0.0001** ^B^
WHR	0.88 ± 0.06	0.86 ± 0.06	−0.02 ± 0.03	0.90 ± 0.03	0.86 ± 0.04	−0.03 ± 0.03	0.86 ± 0.07	0.85 ± 0.08	−0.01 ± 0.03	0.1142 U	**0.0119**	**0.0002**^A^ 0.0842 ^B^
			**X ± SD**			**X ± SD**			**X ± SD**		**TS**	
Relative weight change (%)			−7.34 ± 4.48			−7.07 ± 4.00			−7.57 ± 4.92		0.7214	
Relative change in fat mass (%)			−15.6 ± 9.04			−13.6 ± 7.33			−17.3 ± 10.1		0.1976	
Relative change in BF % (%)			−9.14 ± 6.54			−7.24 ± 4.67			−10.7 ± 7.49		0.0865	

N—Abundance, X—Mean, SD—Standard deviation, TS—Student’s *t* test, U—Mann–Whitney U test, ^O^—Statistic for total subjects, ^A^—Statistic for diet A, ^B^—Statistic for diet B, BF—Body fat, TBW—Total body water, WHR—Waist to hip ratio, BMI—Body mass index.

**Table 4:** Intestinal microbiota of study participants before and after 3 months of dietary change.

Bacteria	Total Respondents N = 42			Diet A N = 19			Diet B N = 23			*p* between the Groups before the Intervention	ANOVA *p* for Interaction	ANOVA *p* for Change over Time
	Before	After	Change	Before	After	Change	Before	After	Change			
	X ± SD	X ± SD	X ± SD	X ± SD	X ± SD	X ± SD	X ± SD	X ± SD	X ± SD			
*Bifidobacterium* spp. (Log CFU/g)	7.45 ± 0.74	7.49 ± 0.78	0.01 ± 0.86	7.47 ± 0.74	7.64 ± 0.86	0.17 ± 0.73	7.43 ± 0.75	7.35 ± 0.70	−0.15 ± 0.97	0.8735 S	0.2609	0.9388 ^O^
*Bacteroides* spp. (Log CFU/g)	8.85 ± 0.47	8.82 ± 0.61	0.00 ± 0.64	8.77 ± 0.42	9.02 ± 0.36	0.25 ± 0.43	8.92 ± 0.52	8.63 ± 0.73	−0.25 ± 0.71	0.3006 S	**0.0123**	**0.0200**^A^ 0.1380 ^B^
*Akkermansia muciniphila* (Log CFU/g)	4.39 ± 1.74	5.22 ± 1.85	0.80 ± 1.73	4.60 ± 1.57	5.39 ± 1.67	0.80 ± 1.67	4.22 ± 1.90	5.06 ± 2.03	0.81 ± 1.83	0.3141 U	0.9865	**0.0071** ^O^
*Faecalibacterium prausnitzii* (Log CFU/g)	8.27 ± 0.53	8.20 ± 0.52	−0.12 ± 0.66	8.26 ± 0.58	8.45 ± 0.33	0.19 ± 0.53	8.27 ± 0.50	7.96 ± 0.57	−0.41 ± 0.65	0.9063 U	**0.0031**	0.1377 ^A^ **0.0102** ^B^
Total bacterial count (Log CFU/g)	9.32 ± 0.25	9.23 ± 0.34	−0.09 ± 0.43	9.24 ± 0.28	9.36 ± 0.18	0.12 ± 0.29	9.39 ± 0.22	9.11 ± 0.42	−0.28 ± 0.46	0.0705 S	**0.0026**	0.0966 ^A^ **0.0126** ^B^

N—Number, X—Mean, SD—Standard deviation, S—Student’s *t* test, U—Mann-Whitney U test, ^O^—Value of the statistic for the total subjects, ^A^—Value of the statistic for diet A, ^B^—Value of the statistic for diet B, CFU—Colony-forming unit.

## Data Availability

The data presented in this study are not publicly available due to confidentiality reasons. These data are available on request from the corresponding author.

## Supplementary Materials

The following supporting information can be downloaded at: https://www.mdpi.com/article/10.3390/ijerph20021348/s1. Table S1. Food intake of female study participants before dietary change. Table S2. Food intake of female study participants before dietary change.
