# Supplementary material for: Effects of a Diet Containing Sources of Prebiotics and Probiotics and Modification of the Gut Microbiota on the Reduction of Body Fat
Source: Int J Environ Res Public Health. 2023 Jan 11;20(2):1348. doi: 10.3390/ijerph20021348 (PMC9859211; doi:10.3390/ijerph20021348)
Supplement: Supplementary file 1 [file ijerph-20-01348-s001.zip › ijerph-2077669-supplementary.pdf]

Table S1. Demographic data and physical activity of study participants before the study.

Sociodemographic factors		Total respondents		Group A		Group B		p
		N	%	N	%	N	%	
Education	Higher	21	50.0	12	63.2	9	39.1	0.3121
	Secondary	11	26.2	3	15.8	8	34.8	
	Vocational	7	16.7	3	15.8	4	17.4	
	Primary	3	7.1	1	5.3	2	8.7	
Employment	Manual worker	13	31.0	6	31.6	7	30.4	0.4472
	White-collar worker	24	57.1	10	52.6	14	60.9	
	Student	3	7.1	2	10.5	1	4.3	
	Unemployed	2	4.8	1	5.3	1	4.3	
Marital status	Not Married	13	31.0	8	42.1	5	21.7	0.1769
	Married	24	57.1	11	57.9	13	56.5	
	Divorcee	4	9.5	0	0.0	4	17.4	
	Widow	1	2.4	0	0.0	1	4.3	
Physical activity frequency	I do not exercise	25	59.5	10	52.6	15	65.2	0.6133
	Once a week	6	14.3	4	21.1	2	8.7	
	2–3 times a week	7	16.7	3	15.8	4	17.4	
	4–5 times a week	1	2.4	1	5.3	0	0.0	
	Daily	3	7.1	1	5.3	2	8.7	
Duration of physical activity	Up to 30 minutes	30	71.4	11	57.9	19	82.6	0.1506
	30–60 minutes	4	9.5	2	10.5	2	8.7	

1–2 hours	8	19.0	6	31.6	2	8.7

N — number, p - chi-squared test.

Table S2. Food intake of female study participants before dietary change.

Products and product groups analysed	TOTAL RESPONDENTS N=42			DIET A N=19	DIET B N=23	Mann-Whitney U test p
	**Me±Q**	**Min**	**Max**	**Me±Q**	**Me±Q**	
CEREAL PRODUCTS [g]	173.6 ± 44.2	92.2	340.5	174.2 ± 25.1	173.0 ± 47.1	0.8596
Groats [g]	0.00 ± 0.00	0.0	33.2	0.00 ± 0.00	0.00 ± 0.00	0.7046
Light bread [g]	93.3 ± 23.3	0.0	250.0	101.4 ± 43.3	90.0 ± 26.7	0.4792
Wholemeal bread [g].	0.0 ± 21.7	0.0	96.7	0.0 ± 26.7	0.0 ± 21.7	0.7810
MILK AND MILK PRODUCTS [g]	234.0 ± 89.2	35.8	698.9	220.0 ± 99.4	251.0 ± 99.7	0.2209
Milk [g]	108.2 ± 48.7	0.0	474.1	108.2 ± 39.1	105.3 ± 58.2	0.8201
Total yoghurts and kefirs [g]	0.5 ± 66.7	0.0	350.0	0.0 ± 66.7	16.7 ± 100.0	0.4484
EGGS [g]	35.4 ± 22.5	0.0	144.7	34.0 ± 22.8	36.7 ± 20.5	0.7235
MEAT, FISH, MEAT PRODUCTS [g]	175.7 ± 45.7	40.0	641.7	187.3 ± 44.6	165.7 ± 61.7	0.6675
Meat [g]	30.9 ± 34.6	0.0	153.0	34.0 ± 25.5	27.2 ± 38.2	0.9095
Poultry [g]	36.3 ± 28.8	0.0	192.5	41.7 ± 26.5	21.3 ± 30.1	0.6675
Fish [g]	0.0 ± 15.8	0.0	76.7	0.0 ± 28.4	0.0 ± 0.0	0.0812
Butter [g]	7.5 ± 6.7	0.0	45.0	5.0 ± 5.8	11.7 ± 9.2	0.1975
VEGETABLES AND FRUIT [g]	286.9 ± 116.9	19.1	1080.5	259.1 ± 82.6	353.9 ± 157.9	0.5956
Vegetables [g]	222.1 ± 93.6	19.1	501.5	192.1 ± 82.9	248.8 ± 85.6	0.2501
Sauerkraut [g]	0.0 ± 1.8	0.0	50.0	0.0 ± 0.0	0.0 ± 11.1	0.3563
Fermented cucumbers [g]	5.1 ± 11.6	0.0	83.3	2.0 ± 6.5	9.0 ± 15.0	0.3182
Fruit [g]	91.7 ± 72.0	0.0	598.8	96.6 ± 63.3	89.5 ± 81.2	0.5611
DRY LEGUME [g]	0.0 ± 0.0	0.0	50.5	0.0 ± 0.0	0.0 ± 0.0	0.8596
SUGARS AND SWEETS [g]	30.2 ± 15.6	0.0	114.9	22.8 ± 13.7	34.7 ± 16.4	0.1975
DRINKS [ml]	1 005 ± 283	133	2966	853 ± 349	1 050 ± 262	0.3369
Coffee, sugar-free infusion [ml]	300.0 ± 133.3	0.0	800.0	200.0 ± 116.7	350.0 ± 150.0	**0.0248**
Tea, sugar-free infusion [ml]	208.3 ± 208.3	0.0	833.3	83.3 ± 208.3	333.3 ± 158.3	0.1327
Highly mineralised water [ml	0.0 ± 0.0	0.0	883.3	0.0 ± 0.0	0.0 ± 0.0	0.8995

N - number, Me - median, Q - quartile deviation, Min - minimum value, Max - maximum value, p - statistical significance value.
